# From the President’s Desk – Part 1, 2023

**DOI:** 10.4102/safp.v65i1.5718

**Published:** 2023-02-21

**Authors:** Robert Mash

**Affiliations:** 1 Division of Family Medicine and Primary Care, Faculty of Medicine and Health Sciences, Stellenbosch University, Cape Town, South Africa

In 2023, the South African Academy of Family Physicians (SAAFP) is preparing for a busy year ahead. In this update from the President’s Desk, I want to outline eight areas that we will be focusing on this year.

In 2022, we started a strong advocacy campaign for the discipline with both the public and private sectors. In the public sector, we published a new position article on the contribution of family physicians to district health services.^1^ The position article was then presented and discussed with stakeholders such as the Deputy Minister of Health, the National Department of Health (those responsible for primary health care and human resources) and the Chair of the Parliamentary Portfolio Committee on Health.^2^ Drs Tasleem Ras and Jenny Nash (directors of the SAAFP) became part of a national group updating policy on district health services. In 2023, we want to focus more on the Provincial Departments of Health and already had a positive engagement with the Department of Health in Mpumalanga. In addition to these engagements, we have published in national media to inform the public.^3^

Dr Sheena Mathew has been leading our initiatives with the private sector and has established a private sector forum so that we can better understand and advocate for the needs of family physicians in private practice. Three key issues are the registration of family physicians as specialists, the remuneration of family physicians and scope of practice, as well as the ability to work in mixed practices and multidisciplinary teams with nonspecialists. We would like to contract with a consultancy to help us address these issues, but we need at least 50 members in private practice to contribute an additional membership levy to enable this. We are also developing a position article on the contribution of family physicians to the private sector to support the conversation with medical schemes and other institutions.

Another initiative will focus on the growing need for skills in point-of-care ultrasound (PoCUS). Dr Shane Murphy is developing a short course on PoCUS, which will equip participants with a broad range of skills, in collaboration with the Global Ultrasound Institute. In addition, Prof. Richard Cooke and Prof. Mergan Naidoo are negotiating with Butterfly and the Bill and Melinda Gates Foundation on an initiative to provide 500 PoCUS machines to public sector maternal care across the country. This should improve maternal, foetal and neonatal outcomes. Please check out our other continuing professional opportunities on the SAAFP website.^4^

The 4th edition of the South African Family Practice Manual is also due to be published in February 2023. The manual covers all the skills needed by a clinician working in primary healthcare or district health services. Students and practitioners will find this a valuable resource, particularly registrars in family medicine, medical officers, general practitioners and family physicians, as well as clinical associates and nurse practitioners. We have included new sections on rehabilitation and palliative care, as well as new chapters on, for example, sexual health, PoCUS, mentoring and helping the learner in difficulty.

The National Family Practitioners’ Conference will be held from 17 August 2023 to 19 August 2023 at the Wanderers in Johannesburg.^5^ This year, our colleagues in KwaZulu-Natal are leading the organisation, and the theme is ‘Integrating primary care – creating a more connected health and care system’. As usual, the conference will be a mix of practical workshops, plenary talks, seminars and research presentations. It will provide a chance to update clinically, engage in dialogue on the key issues and network with colleagues. We hope to see you there.

The World Organisation of Family Doctors (Wonca) will hold its global conference in Sydney, Australia, from 26 to 29 October 2023.^6^ The SAAFP represents South Africa on Wonca and will attend the Wonca World Council as well as participate in many of the Working Parties. Wonca has member organisations from 110 countries, covering nearly 90% of the world’s population. The theme of the conference is ‘Recovery, reconnection and revival – a celebration of primary care’.

The education and training committee of the SAAFP continues to focus on workplace-based training and assessment. Prof. Louis Jenkins is leading a group to define entrustable professional activities (EPAs) that will guide the future assessment of registrars. The SAAFP has also led the way in implementing an e-portfolio across the country to help document learning in the workplace. We will also be running our training of clinical trainers course later in the year to improve the quality of clinical training, under the leadership of Prof. Hanneke Brits and her team. I hope that we will also introduce accreditation of clinical trainers during 2023.

Finally, we will elect a new council and a new executive later this year. You can view the current council on our website.^7^ The request for nominations will be distributed in February. The council includes members in clinical practice (private and public sector), academics, rural doctors, associate and student members. If you are in clinical practice and would like to contribute to the SAAFP, then please consider putting yourself forward for election.
